# Hemorrhagic Events Associated With Direct Oral Anticoagulants: Frequency and Management

**DOI:** 10.7759/cureus.98282

**Published:** 2025-12-02

**Authors:** Mohamed Ztati, Oussama Zaanouny, Ayoub Nasim, Mustapha El Hattaoui

**Affiliations:** 1 Cardiology, Hospital Arrazi, Centre Hospitalo-Universitaire (CHU) Mohammed VI, Université Cadi Ayyad (UCA) Faculty of Medicine and Pharmacy in Marrakech, Marrakech, MAR

**Keywords:** antithrombotic therapy, bleeding management, direct-acting oral anticoagulants (doacs), hemorrhagic complications, oral anticoagulants

## Abstract

Background

Direct oral anticoagulants (DOACs) have transformed anticoagulation strategies, yet bleeding complications remain a significant concern, particularly in elderly patients with polypharmacy.

Methodology

In this three-year, cross-sectional study conducted at a tertiary cardiology center in Morocco, we analyzed 35 cases of non-traumatic DOAC-related bleeding.

Results

The mean patient age was 67.5 years, and 89% were on multiple medications. Rivaroxaban was the most prescribed DOAC (74%), and gastrointestinal bleeding was the most common presentation (46%), followed by intracerebral hemorrhage and epistaxis (14% each). Major bleeding occurred in 34% of cases; however, no specific reversal agents were used. Despite resource limitations, outcomes were favorable in 94% of patients, with only two deaths recorded.

Conclusions

These real-world findings highlight the importance of individualized DOAC management, structured medication reviews, and improved access to reversal agents. In resource-limited settings, standardized protocols and clinician training are crucial to bridge the gap between guideline recommendations and everyday clinical practice.

## Introduction

Anticoagulants are widely prescribed to prevent thromboembolic events, particularly in non-valvular atrial fibrillation, mechanical heart valves, and venous thromboembolism. However, their use carries a significant risk of bleeding, regardless of the agent. The increasing prescription of anticoagulants, driven by population aging and the growing adoption of direct oral anticoagulants (DOACs), has led to a rise in anticoagulant-related hemorrhagic events. These events represent a leading cause of drug-related hospitalizations and are associated with substantial morbidity and mortality. Notably, a recent study reported that hospitalization rates for bleeding were nearly four times higher in DOAC users compared with non-users (27.5 vs. 6.7 per 1,000 beneficiaries) [1]. Furthermore, up to 15% of anticoagulated patients require surgery or invasive procedures within a year, highlighting the clinical importance of effective bleeding management. Optimizing care for these patients is therefore a priority, underscoring the need for standardized reversal strategies in emergency settings [2].

## Materials and methods

The primary objective of this study was to determine the frequency of hemorrhagic events associated with DOACs in the Cardiology Department of Arrazi Hospital, Mohammed VI University Hospital Center, Marrakech, and to establish standardized protocols for their management. In addition, the study aimed to identify cardiovascular risk factors and predictors of hemorrhagic complications, to describe the most frequent hemorrhagic events, including their localization and severity, and to propose standardized therapeutic strategies to optimize patient care.

This work was designed as a descriptive, cross-sectional study conducted over a three-year period, from January 2022 to January 2025, in the Cardiology Department of Arrazi Hospital, Mohammed VI University Hospital Center, Marrakech. The study population included all patients receiving DOAC therapy who presented to the Emergency Department or were referred to our institution with external or internal hemorrhagic manifestations. Patients with bleeding events related to trauma, as well as those with absent or incomplete medical records, were excluded from the analysis. A total of 35 patients who experienced non-traumatic external or internal hemorrhagic events while receiving DOAC therapy were included.

Data were collected using a structured form integrating demographic characteristics, cardiovascular risk factors, past medical and surgical history, clinical presentation at admission, paraclinical and radiological findings, and therapeutic management. Medical records were reviewed to ensure adequate follow-up during hospitalization, and the hospital information system (Ho-six.NET) was used to extract complementary data, including laboratory results and length of stay.

Bleeding severity was classified according to the International Society on Thrombosis and Haemostasis definition of major bleeding, which includes any fatal bleeding, symptomatic bleeding in a critical area or organ (e.g., intracranial, intraspinal, intraocular, retroperitoneal, intra-articular, pericardial, or intramuscular with compartment syndrome), or bleeding causing a fall in hemoglobin of ≥2 g/dL or requiring transfusion of ≥2 units of blood. For additional consistency, events were also reviewed using the Bleeding Academic Research Consortium classification, and all major bleeds in this study corresponded to BARC type 3a-3c.

All procedures were conducted in accordance with ethical standards. Patient anonymity was preserved, and the confidentiality of medical information was strictly maintained throughout the study.

## Results

Over the three-year study period (January 2022 to January 2025), 1,823 patients received a DOAC in the Cardiology Department of Mohammed VI University Hospital in Marrakech. Among them, 35 (1.9%) patients developed a hemorrhagic complication requiring hospitalization, while 1,788 (98.1%) remained free of bleeding. Therefore, DOAC-related hemorrhagic events represented 1.9% of all cardiology admissions during the study period. The mean age of bleeding patients was 67.5 ± 10.8 years, with a slight male predominance (19 males, 54%). The most frequent cardiovascular risk factors were sedentary lifestyle (21 patients, 60%), hypertension (17, 49%), smoking (15, 43%), and diabetes or dyslipidemia (12, 34% each). The mean body weight was 71 ± 9 kg, serving as an indirect indicator of frailty. The mean serum creatinine was 11.8 ± 4.6 mg/L, and the mean estimated glomerular filtration rate (eGFR) (MDRD formula) was 65.1 ± 18.7 mL/minute/1.73 m², with 11 (31%) patients showing moderate renal impairment (eGFR < 60 mL/minute/1.73 m²). The mean baseline hemoglobin was 10.6 ± 2.3 g/dL, and 26 (75%) patients were anemic at admission. The mean platelet count was 228 ± 67 × 10⁹/L. Regarding concomitant treatments, 31 (89%) patients were polymedicated, including antiplatelet agents in seven (20%) cases, non-steroidal anti-inflammatory drugs (NSAIDs) in three (9%) cases, and amiodarone in one (3%) nonsteroidal anti-inflammatory drugs. The mean HAS-BLED score was 2.3 ± 0.9, with five (14%) patients classified as high-risk (score ≥3). Non-valvular atrial fibrillation was the leading indication for anticoagulation (17 patients, 49%), followed by valvular disease (10 patients, 28%) and coronary artery disease (4 patients, 11%) (Table 1).

**Table 1:** Baseline characteristics of patients with DOAC-related hemorrhagic events (N = 35). DOAC = direct oral anticoagulant; NSAID = non-steroidal anti-inflammatory drug

Characteristic	Value (N = 35)	χ²/t-value	P-value
Age (years, mean ± SD)	67.5 ± 10.8	t = 1.43	0.16
Male sex	19 (54%)	χ² = 0.32	0.57
Body weight (kg)	71 ± 9	—	—
Sedentary lifestyle	21 (60%)	χ² = 0.45	0.50
Hypertension	17 (49%)	χ² = 0.89	0.34
Diabetes mellitus	12 (34%)	χ² = 0.22	0.64
Dyslipidemia	12 (34%)	χ² = 0.22	0.64
Creatinine (mg/L)	11.8 ± 4.6	—	—
eGFR (mL/minute/1.73 m²)	65.1 ± 18.7	—	—
Hemoglobin (g/dL)	10.6 ± 2.3	—	—
Platelets (×10⁹/L)	228 ± 67	—	—
Antiplatelet therapy	7 (20%)	—	—
NSAID use	3 (9%)	—	—
HAS-BLED score (mean ± SD)	2.3 ± 0.9	—	—
High-risk patients (HAS-BLED ≥3)	5 (14%)	—	—

Rivaroxaban was the most frequently prescribed DOAC (26 patients, 74%), followed by apixaban (9, 26%). Regarding dosing, 29 (83%) patients were treated with a standard full therapeutic dose, while six (17%) patients received a reduced dose adjusted for renal function, advanced age, or low body weight, in accordance with current guideline recommendations. The gastrointestinal tract was the predominant bleeding site (16 patients, 46%), followed by intracerebral hemorrhage (5, 14%) and epistaxis (5, 14%) (Table 2).

**Table 2:** DOAC distribution and bleeding sites (N = 35). DOAC = direct oral anticoagulant

Variable	Value (N = 35)	χ² value	P-value
Rivaroxaban use	26 (74%)	—	—
Apixaban use	9 (26%)	—	—
Gastrointestinal bleeding	16 (46%)	χ² = 5.42	0.02
Intracerebral hemorrhage	5 (14%)	χ² = 0.88	0.34
Epistaxis	5 (14%)	χ² = 0.88	0.34

Major bleeding occurred in 12 (34%) patients, of whom seven (20%) required transfusion and three (9%) underwent emergency neurosurgical evacuation. No reversal agents were used. Anticoagulation was discontinued in all cases, with temporary low-molecular-weight heparin bridging when necessary. The median hospital stay was 7 ± 2.3 days. Two (6%) patients died, one from refractory hemorrhagic shock and one following neurosurgical complications, while 33 (94%) patients had a favorable outcome (Table 3).

**Table 3:** Outcomes, interventions, and anticoagulant dosing among patients with DOAC-related bleeding (N = 35). DOAC = direct oral anticoagulant

Outcome/Intervention	Value (N = 35)	χ²/t-value	P-value
Major bleeding	12 (34%)	χ² = 3.12	0.08
Transfusion required	7 (20%)	χ² = 4.05	0.04
Neurosurgical evacuation	3 (9%)	χ² = 0.55	0.46
Hospital stay (days, mean ± SD)	7 ± 2.3	t = 2.05	0.047
Mortality	2 (6%)	χ² = 0.92	0.34
DOAC dose			
Full therapeutic dose	29 (83%)	—	—
Reduced dose	6 (17%)	—	—

## Discussion

An important observation of our study is the overwhelming prevalence of polypharmacy, observed in 89% of patients, which aligns with the findings of Cuker et al. [3], who highlighted the synergistic bleeding risk when DOACs are combined with agents such as antiplatelets, NSAIDs, or amiodarone. These data underscore the urgent need for structured medication reviews, particularly in elderly patients frequently exposed to multiple interacting drugs without systematic risk assessment. A major concern is the lack of access to specific reversal agents, such as idarucizumab or andexanet alfa, which were not administered to any patients despite major bleeding occurring in over one-third of cases, reflecting the situation in many low- and middle-income countries where cost and availability restrict routine use despite guideline recommendations [4]. Consequently, clinicians had to rely on general supportive care, temporary discontinuation of therapy, and, in some cases, LMWH bridging. While the observed mortality rate of 6% was lower than reported in some multicenter studies, the absence of reversal agents may expose patients to preventable complications or death in severe scenarios.

At a systems level, these findings emphasize the need for institutional protocols tailored to DOAC emergencies, including local access to reversal agents, rapid laboratory assays for coagulation parameters, and standardized decision-making pathways, complemented by clinician training in bleeding risk evaluation tools such as HAS-BLED, which remain underutilized despite proven clinical utility [5,6]. Importantly, international and European guidelines, including the European Society of Cardiology, European Heart Rhythm Association, and French Working Group on Perioperative Hemostasis (Groupe d'Intérêt en Hémostase Périopératoire, GIHP) recommendations, already provide structured management algorithms for DOAC-related bleeding, outlining stepwise approaches to evaluation, classification (major vs. non-major), and treatment based on hemodynamic status, bleeding site, and DOAC pharmacokinetics [7-9]. Our study supports and aligns with these algorithms, demonstrating their applicability in real-world settings, while also highlighting practical limitations in resource-constrained environments where drug availability and laboratory capacity may limit full implementation.

This study also raises important considerations regarding anticoagulation reinitiation: in our cohort, most patients were restarted on DOAC therapy after stabilization, but such decisions must be individualized, with emerging evidence suggesting that resumption within 7-14 days post-bleed may reduce long-term thrombotic risk without significantly increasing recurrent bleeding, although high-quality data across diverse populations remain limited [5]. Pending updated formal guidelines, the management of DOAC-related bleeding may follow adapted strategies based on national recommendations for VKAs [7], expert proposals from the GIHP [8], and current evidence on reversal agents. Immediate discontinuation of the anticoagulant is the first step, regardless of plasma concentration, given the short half-life of DOACs.

Bleeding should be classified as major or non-major, with major bleeding involving critical sites (e.g., intracranial, retroperitoneal), hemodynamic compromise, transfusion of ≥2 units of red blood cells, or a hemoglobin drop ≥2 g/dL, while non-major bleeding refers to controllable hemorrhage in non-critical locations. Initial evaluation should consider age, weight, type and timing of the last DOAC dose, indication, hemodynamic status, and comorbidities, and laboratory workup should include prothrombin time, activated partial thromboplastin time, renal and hepatic function, complete blood count, and, when available, DOAC-specific assays such as anti-Xa activity or thrombin time [9].

In life-threatening hemorrhage, particularly involving critical organs (e.g., brain, spinal cord, eye) or associated with hemorrhagic shock, reversal should be initiated without delay, even before plasma DOAC levels are available. For other major bleeding events, therapeutic decisions may be guided by DOAC concentrations, with levels <30 ng/mL indicating that the anticoagulant effect is unlikely and reversal is not indicated, whereas levels ≥30 ng/mL with persistent bleeding despite supportive and local measures justify administration of reversal agents [9]. Minor or non-severe bleeding should be managed symptomatically without reversal agents, including identification of risk factors for DOAC accumulation, such as renal or hepatic dysfunction and interacting drugs. Severe renal impairment (creatinine clearance (CrCl) <30 mL/minute) or hepatic failure contraindicate DOAC use and require therapy modification, and plasma concentrations >400 ng/mL, particularly with renal dysfunction, may warrant skipping a dose to allow clearance, followed by reassessment and reinforcement of patient education.

**Figure 1:**
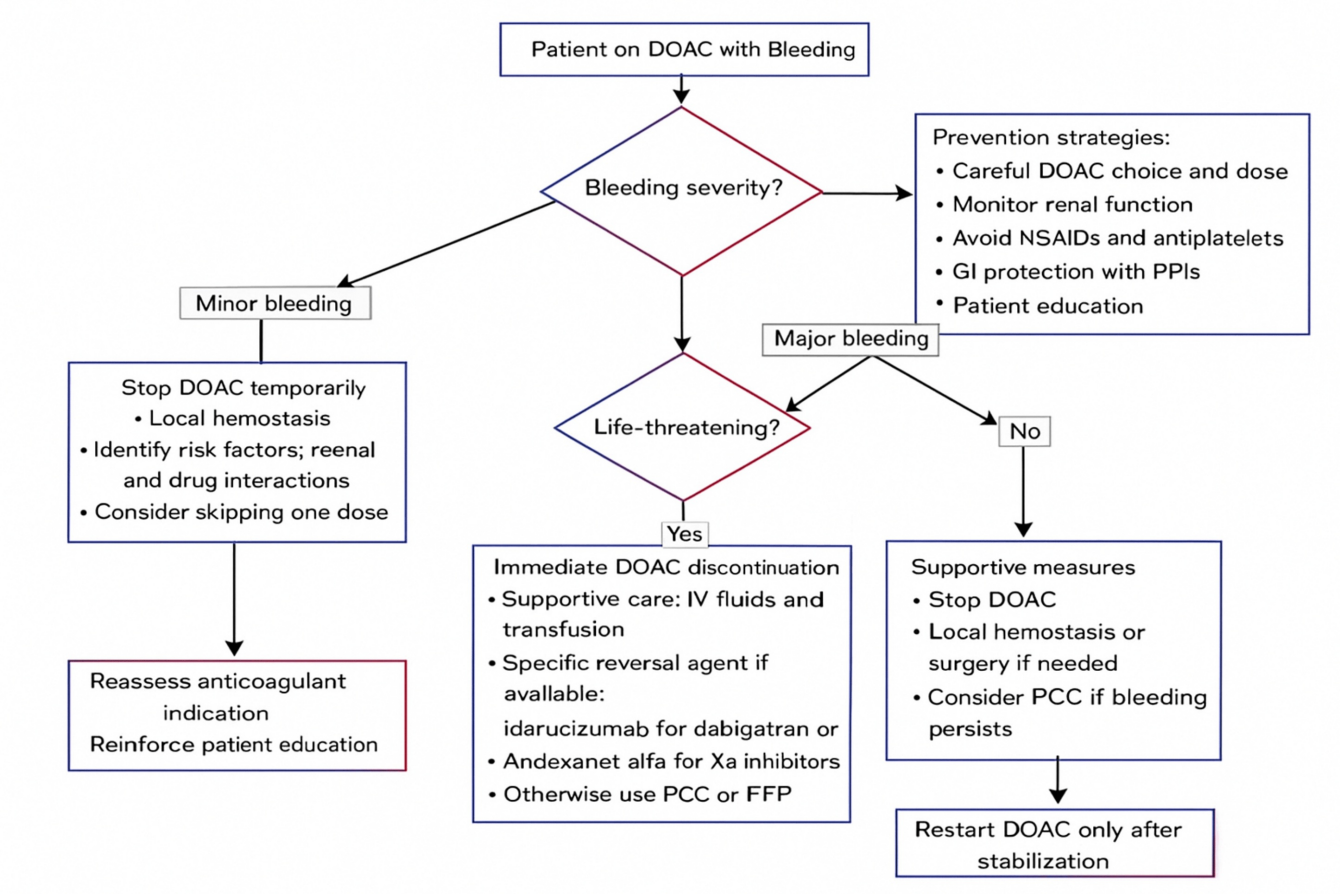
Management algorithm for bleeding events in patients on DOACs. This figure was generated by the authors. DOAC = direct oral anticoagulant; NSAID = non-steroidal anti-inflammatory drug; GI = gastrointestinal; PPI = proton-pump inhibitor; FFP = fresh frozen plasma; PCC = prothrombin complex concentrate; ICU = intensive care unit

Prevention of bleeding events requires a comprehensive, individualized approach combining clinical, pharmacological, and educational strategies, including careful drug choice and dose adjustment based on age, renal function, weight, comorbidities, and bleeding risk, with apixaban preferred in patients at high risk of gastrointestinal bleeding due to its better safety profile [10,11] and dose reductions applied for age ≥80 years, weight ≤60 kg, or CrCl <30 mL/minute depending on the agent. Regular monitoring of renal function every 6-12 months, or more frequently if unstable, and complete blood counts to detect occult anemia are recommended, and poorly controlled hypertension (>160 mmHg) should be optimized to reduce intracranial bleeding risk [10]. Avoidance of drug interactions is essential, with NSAIDs, antiplatelets, corticosteroids, selective serotonin reuptake inhibitors, and potent P-gp or CYP450 inhibitors (e.g., amiodarone, verapamil, rifampicin) used only when strictly indicated due to their additive bleeding risk and potential to increase DOAC plasma concentrations [11]. Gastrointestinal protection with proton pump inhibitors is strongly recommended in at-risk patients, particularly those with prior ulcers or on combined DOAC and NSAID/aspirin therapy [12]. Finally, patient education is critical, emphasizing adherence; avoidance of self-medication (especially NSAIDs)l recognition of warning signs such as unexplained bruising, hematuria, rectal bleeding, or fatigue; and carrying an anticoagulant card for emergencies [13]. Figure 1 presents a management algorithm to manage bleeding events in patients on DOACs.

## Conclusions

DOACs have revolutionized stroke prevention in atrial fibrillation and the management of thromboembolic disease, yet the risk of bleeding remains clinically significant. While expert guidelines recommend specific assays for monitoring and managing DOAC-associated hemorrhage, their limited availability often necessitates reliance on conventional hemostatic tests. The introduction of idarucizumab has provided an effective reversal strategy for dabigatran, whereas prothrombin complex concentrates continue to play a central role in mitigating bleeding from factor Xa inhibitors until targeted antidotes become more accessible. Optimizing DOAC therapy requires a multidisciplinary strategy emphasizing patient education, adherence, early recognition of adverse events, and rapid response to complications to balance efficacy with safety.
